# Skeletal and Dentoalveolar Components of Maxillary Expansion: A Systematic Review of Post-Treatment Stability

**DOI:** 10.3390/children13081057

**Published:** 2026-08-08

**Authors:** Niccolò Cenzato, Alessia Tremolada, Alessandra Comparini, Fausto Zamparini, Cinzia Maspero

**Affiliations:** 1 Department of Biomedical, Surgical and Dental Sciences, University of Milan, 20122 Milan, Italy; niccolo.cenzato@unimi.it (N.C.); alessia.tremolada@studenti.unimi.it (A.T.); cinzia.maspero@unimi.it (C.M.); 2 Fondazione IRCCS Ca’ Granda Ospedale Maggiore Policlinico, 20122 Milan, Italy; 3 Dipartimento di Scienze Biomediche e Neuromotorie (DIBINEM), Alma Mater Studiorum Università di Bologna, 40126 Bologna, Italy

**Keywords:** maxillary expansion, rapid palatal expansion (RPE), bone-borne expander, tooth-borne expander, hybrid maxillary expansion, skeletal stability, dentoalveolar effects, midpalatal suture, orthodontic relapse, post-treatment follow-up

## Abstract

**Highlights:**

**What are the main findings?**

- Bone-supported expanders may increase the initial skeletal contribution, but superior long-term stability was not demonstrated.
- A five-year relapse was identical with tooth-borne and hybrid expansion and appeared to be mainly related to dentoalveolar changes, while skeletal expansion remained comparatively stable.

**What are the implications of the main findings?**

- A greater initial skeletal effect should not be considered evidence of greater long-term stability.
- Appliance selection should be individualized according to skeletal maturity and periodontal risk.

**Abstract:**

**Background:** Maxillary transverse deficiency is a common skeletal discrepancy in orthodontic patients that often requires expansion therapy for correction. Failure to address this condition during growth may compromise the orthopedic prognosis, frequently necessitating surgically assisted rapid palatal expansion (SARPE) or other orthognathic surgical procedures after skeletal maturity. **Methods:** The studies in this systematic review were selected according to predefined inclusion criteria. Randomized controlled trials, controlled clinical studies, and cohort studies published within the last 10 years with a follow-up ≥ 6 months were included. The databases PubMed, Scopus, and Embase were searched. Orthodontic outcomes included midpalatal suture opening, skeletal transverse changes, intermolar width variations, dental tipping, and long-term stability. Thirteen studies met the eligibility criteria. Risk of bias was assessed using the RoB 2 and the Newcastle–Ottawa Scale. **Results:** All orthodontic devices produced significant transverse expansion in the short term. Bone-borne and hybrid systems showed a greater initial skeletal component, with greater expansion at the maxillary and nasal basal levels and limited relapse over time. Tooth-borne expansion was associated with greater dental tipping and reduction in buccal bone thickness, with partial recovery during retention. Over time, loss of expansion mainly affected the dentoalveolar component, whereas the skeletal component remained more stable. Long-term data (≥3–5 years) remain limited but suggest that relapse is predominantly related to dental uprighting rather than a true loss of skeletal base width. **Conclusions:** Long-term transverse stability improves when expansion is predominantly skeletal. Bone-supported and hybrid appliances may produce a greater initial skeletal contribution and reduce dental tipping in some clinical settings; however, current evidence does not demonstrate superior long-term stability compared with tooth-borne expansion. Additional prospective orthodontic studies with extended follow-up, particularly without prolonged retention, are required.

## 1. Introduction

Transverse maxillary deficiency is one of the most common skeletal discrepancies encountered in orthodontic patients, particularly during childhood and adolescence [1]. If left untreated during growth, it may result in posterior crossbite, dental crowding, functional mandibular deviation, facial asymmetry, and impaired occlusal development. Early orthopedic correction is therefore considered essential to restore transverse skeletal relationships and improve long-term functional and occlusal outcomes, reducing the likelihood of requiring surgically assisted rapid palatal expansion (SARPE) or orthognathic surgery after skeletal maturity [2,3,4].

Maxillary expansion is the treatment of choice for correcting transverse maxillary deficiency in growing patients. The biological response to expansion depends mainly on appliance design and the patient’s skeletal maturity [2]. Conventional tooth-borne rapid maxillary expansion (RME), bone-borne or miniscrew-assisted rapid palatal expansion (MARPE), hybrid expanders, and surgically assisted rapid palatal expansion (SARPE) represent the principal therapeutic approaches currently available [3,4].

Skeletal expansion is primarily obtained through opening of the midpalatal suture, particularly in growing patients, whereas dentoalveolar expansion mainly results from dental tipping, alveolar bone remodeling, and orthodontic tooth movement [4,5,6]. The relative contribution of these two components varies according to the type of appliance, the patient’s skeletal maturity, and the treatment protocol [3,6]. Although direct evaluation of skeletal changes by repeated CBCT examinations is limited by ethical considerations, several studies have demonstrated that skeletal anchorage may increase the orthopedic component of expansion while reducing undesirable dentoalveolar side effects [3,7,8,9].

Although maxillary expansion has been extensively investigated, the long-term stability of the achieved correction remains one of the most clinically relevant issues. A comprehensive evaluation of craniofacial morphology during growth is essential for accurate diagnosis and treatment planning, as occlusal alterations and transverse maxillary deficiencies may be associated with changes in both skeletal and soft-tissue facial characteristics, as well as with functional factors such as upper airway obstruction. Previous studies have investigated facial soft-tissue morphology, maxillomandibular relationships, and the effects of transverse maxillary expansion on craniofacial structures, highlighting the complex interaction between occlusion, growth patterns, and orthodontic treatment outcomes [10,11,12,13]. Following active expansion, relapse may occur because of dentoalveolar remodeling, periodontal adaptation, retention protocols, or incomplete skeletal consolidation. Consequently, identifying the biological and treatment-related factors influencing post-treatment stability is essential for selecting the most appropriate expansion appliance and retention strategy [14,15,16,17,18]. Several clinical studies and systematic reviews have evaluated the effectiveness of different maxillary expansion appliances, particularly regarding the amount of transverse expansion achieved, airway changes, and short-term treatment outcomes [15,16,17,18]. More recently, systematic reviews have specifically compared MARPE with conventional tooth-borne expanders [15,16]. However, the available evidence remains heterogeneous because of differences in study design, patient age, imaging modalities, retention protocols, and outcome assessment [17,18].

Importantly, despite the availability of several systematic reviews evaluating maxillary expansion, none has specifically focused on differentiating the long-term stability of the skeletal and dentoalveolar components of expansion. Most reviews evaluate transverse expansion as a single outcome, limiting the possibility of identifying the biological source of relapse. Clarifying this issue is clinically relevant because it may influence appliance selection, retention protocols, and long-term treatment prognosis [17,18]. Therefore, the aim of the present systematic review was to evaluate the available evidence regarding the long-term stability of maxillary expansion by separately analyzing skeletal and dentoalveolar outcomes. Specifically, the objectives were to evaluate the persistence of the achieved expansion over time, compare the long-term stability of different expansion approaches, and provide clinically relevant evidence to support appliance selection and post-treatment retention strategies.

## 2. Materials and Methods

This systematic review was conducted and reported in accordance with the Preferred Reporting Items for Systematic Reviews and Meta-Analyses (PRISMA) 2020 statement.

### 2.1. Eligibility Criteria

To perform this systematic review, inclusion and exclusion criteria were established.

*Inclusion criteria*: The following study designs were selected: randomized controlled trials (RCTs), controlled clinical trials (CCTs), prospective/retrospective cohorts, and case series with N ≥ 10. In the included studies, patients were required to have at least 6 months of observation after active expansion, including, when applicable, the retention phase. Outcomes measured at the end of retention were analyzed separately from post-retention observations and were interpreted as reflecting post-treatment maintenance rather than true long-term stability. Only follow-up assessments performed after retention removal were considered indicative of true long-term stability. Studies evaluating specific interventions were selected, including rapid palatal expansion tooth-borne (RPE), MARPE (bone-borne/miniscrew), surgically assisted rapid maxillary expansion (SARME), and hybrid expanders. Studies were included if they provided quantitative data on skeletal and/or dentoalveolar effects of maxillary expansion, even when expansion was part of a combined or adjunctive treatment, provided that expansion-related outcomes could be identified and interpreted. The population had to include patients with transverse maxillary deficiency (children/adolescents/adults; non-syndromic). Measurement methods considered were CBCT/cephalometric analyses for skeletal evaluation and CBCT/3D models/digital casts for dentoalveolar evaluation. No language restrictions were applied. Studies published in the last 10 years were included.

*Exclusion criteria*: Studies with follow-up less than 6 months or only immediate measurements were excluded, as well as studies with data not distinguishable between skeletal and dentoalveolar evaluations. Single case reports, editorials, narrative reviews, conference abstracts, and syndromic populations/complex cleft palate cases (unless separate and comparable subgroups were available) were excluded. Studies evaluating interventions not involving maxillary expansion (e.g., aligners only without RME) or combined treatments preventing attribution of the effect were also excluded.

### 2.2. Information Sources

For data retrieval, the following databases were consulted: Scopus, Embase, and PubMed. All searches were performed on 19 October 2025. The review protocol was registered in the International Prospective Register of Systematic Reviews (PROSPERO; registration number CRD420261298117) on 2 February 2026. All database searches were performed on 19 October 2025. The review protocol was subsequently registered in the International Prospective Register of Systematic Reviews (PROSPERO; CRD420261298117) on 2 February 2026. Therefore, protocol registration occurred after completion of the initial database search.

### 2.3. Search Strategy

The search strategy included the development of complete and comprehensive search strings for each database, incorporating the inclusion criteria (Table 1). Boolean operators and MeSH terms were used.

**Figure 1:**
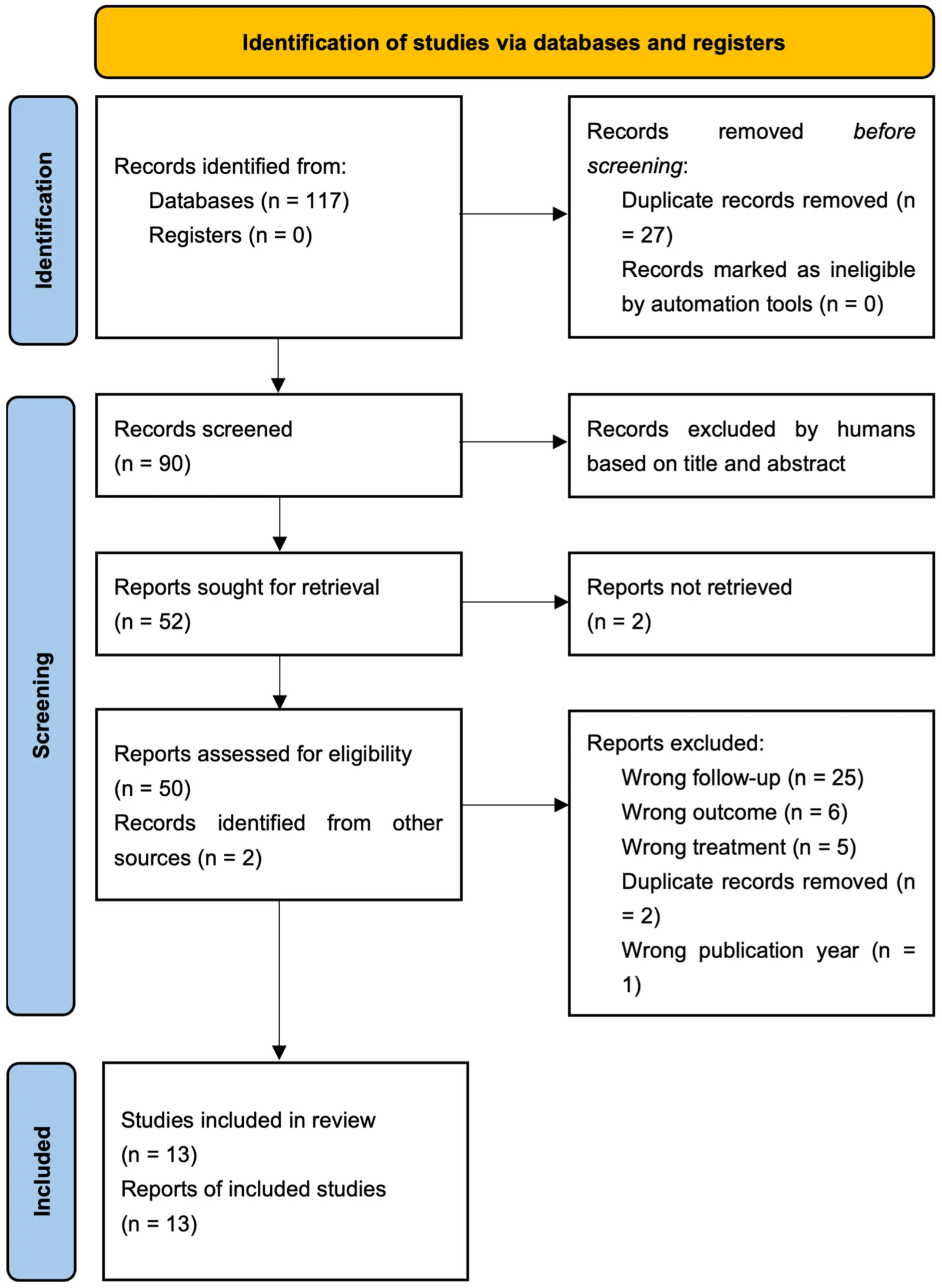
PRISMA flow diagram illustrating the study selection process.

Study selection was performed by two independent reviewers (A.C. and A.T.); any discrepancies or doubts were resolved through consultation with a third reviewer (N.C.). During the selection process, particular attention was given to whether the reported outcomes could be attributed to maxillary expansion. The study selection process is summarized in the PRISMA flow diagram (Figure 1).

In the first phase, starting from a total of 117 records, 27 duplicate studies were identified and removed using the Rayyan software (Rayyan System Inc., Cambridge, MA, USA, web version accessed October 2025), retaining for each duplicate the version considered most appropriate. Subsequently, a preliminary screening of the remaining 90 articles was performed by reading titles and abstracts, which led to the exclusion of 38 studies.

For the remaining 52 articles, the full text was sought. Two articles were not available in full text and were therefore excluded. Full-text evaluation then focused mainly on follow-up duration, with 25 studies excluded because they did not meet the minimum required duration. In addition, 5 articles were excluded because they evaluated treatments not compliant with the inclusion criteria, and a further 6 articles were excluded due to the presence of non-relevant outcomes.

During this phase, two additional duplicate articles not previously detected by Rayyan were also identified; for each of them, the most appropriate version was retained. One article that did not fall within the considered publication time range was also excluded.

The search strategy was re-examined and refined to improve sensitivity and ensure comprehensive identification of relevant studies. In addition to the electronic database search, a manual screening of reference lists was performed, identifying two additional studies [19,20] that met the predefined inclusion criteria. These studies have been included in the revised manuscript to improve the completeness of the evidence base. Therefore, the final number of studies included in the systematic review was 13 articles, listed in Table 2. Studies analyzing only one type of maxillary expansion were also included; the results of these studies were subsequently compared with those of other publications. The observation period following active expansion could include the retention phase, but outcomes recorded at the end of retention were considered separately from post-retention outcomes and were not interpreted as true long-term stability. Accordingly, studies were included if they reported at least one post-expansion assessment with a minimum observation period of 6 months. When the last available assessment corresponded to the end of retention, it was interpreted as post-treatment maintenance rather than true long-term stability.

Because age and skeletal maturity may substantially influence the orthopedic and dentoalveolar response to maxillary expansion, the included studies were stratified according to the skeletal maturity of the participants. Studies involving prepubertal or peripubertal patients were considered separately from those involving late adolescents and skeletally mature adults.

### 2.4. Data Items

Data were extracted independently by two reviewers using a standardized data extraction form.

For each study, the following were recorded:

- Sample characteristics (age, sample size, clinical conditions);
- Type of appliance (bone-borne, tooth-borne, hybrid);
- Diagnostic methodologies used (CBCT, lateral cephalogram, digital models);
- Retention duration and post-retention follow-up, when reported;
- Skeletal outcomes (suture opening, transverse maxillary changes);
- Dentoalveolar outcomes (intermolar width variations, dental tipping, buccal cortical bone thickness);
- Stability indicators and relapse rate.

Secondary outcomes included sagittal and vertical skeletal changes when available.

### 2.5. Risk-of-Bias Assessment

Risk-of-bias assessment was conducted according to the type of each study, using internationally recognized methodological tools.

The two reviewers performed the assessments, and any discrepancies were resolved through discussion until consensus was reached.

Randomized controlled trials were assessed using the Cochrane Risk of Bias 2 tool for the effect of assignment to the intervention. The assessment considered five domains: bias arising from the randomization process, bias due to deviations from the intended interventions, bias due to missing outcome data, bias in the measurement of the outcome, and bias in the selection of the reported result. Each domain and the overall risk of bias were classified as “low risk”, “some concerns”, or “high risk”.

RoB 2 assessments referred to the principal skeletal or dentoalveolar outcome relevant to the present review for each randomized trial.

Regarding non-randomized and cohort studies, methodological quality was assessed using the Newcastle–Ottawa Scale (NOS), which evaluates three categories: Selection (4 points), Comparability (2 points), and Outcome (3 points).

Observational studies showed limitations mainly related to comparability and the absence of untreated control groups. The certainty of evidence was assessed using the GRADE approach for four main outcome categories: skeletal transverse changes, dentoalveolar changes and dental tipping, periodontal and alveolar bone changes, and post-treatment stability or relapse. The assessment considered risk of bias, inconsistency, indirectness, imprecision, and publication bias. Because statistical pooling was not considered appropriate, the evaluation was based on the structured narrative synthesis and was performed separately for clinically comparable outcomes. Certainty of evidence was classified as high, moderate, low, or very low. Two reviewers independently performed the assessments, and disagreements were resolved through discussion and consensus.

Publication bias was considered qualitatively within the GRADE assessment. Formal assessment using funnel plots or regression-based tests was not performed because no meta-analysis was conducted and fewer than 10 studies contributed to each clinically homogeneous comparison.

### 2.6. Effect Measures

The effect measures considered in the present review included skeletal and dentoalveolar variations following maxillary expansion. In particular, transverse maxillary changes, opening of the midpalatal suture, modifications in intermolar width, and dental tipping were analyzed. Where reported by the original studies, effect estimates and corresponding 95% confidence intervals were extracted and presented alongside *p* values.

### 2.7. Synthesis Methods

Due to the high clinical, methodological, and instrumental heterogeneity among the included studies, a meta-analysis was not considered appropriate. A narrative synthesis was therefore conducted.

To improve comparability across studies, outcomes were conceptually interpreted according to standardized timepoints: T0 (pre-treatment), T1 (immediate post-expansion), T2 (end of retention), and T3 (post-retention follow-up where available).

Outcomes were analyzed separately when reported during retention (T1–T2) and after retention (T2–T3), when data were available.

Studies were grouped according to the following:

- Type of appliance (bone-borne vs. tooth-borne);
- Skeletal outcomes;
- Dentoalveolar outcomes;
- Post-treatment stability.

In addition, results were interpreted in light of major clinical and methodological effect modifiers, including age/skeletal maturity, surgical versus non-surgical approach, protocol characteristics, imaging modality, and retention regimen.

Another relevant source of heterogeneity is the amount and rate of activation, which varied substantially across studies and may have influenced both the magnitude of expansion and the relative skeletal versus dentoalveolar contribution.

## 3. Results

The characteristics and principal findings of the included studies are summarized in Table 3. Table 3 provides a structured overview of study design and clinical heterogeneity, including age and skeletal maturity, treatment protocol, measurement method, retention, and follow-up timepoints. The characteristics and principal findings of the included studies are summarized in Table 4. Study design, patient age and skeletal maturity, treatment protocol, measurement method, retention regimen, and follow-up duration varied across the included studies. The risk-of-bias assessments are presented in Table 4. Overall, the randomized trials predominantly raised some concerns. The most frequent concern involved the selection of the reported result, mainly because of the absence of a publicly available prespecified analysis plan or prospective registration in some trials. Additional concerns regarding randomization, deviations from the intended interventions, missing outcome data, or outcome measurement were identified in individual studies. No numerical summary score was calculated for randomized trials.

The Newcastle–Ottawa Scale assessments of the non-randomized and cohort studies ranged from 7 to 8 points out of 9, indicating a low-to-moderate risk of bias.

The study by Altieri et al. (2022) [26] showed the highest methodological rigor, obtaining a score of 8/9; sample selection and full group comparability resulted in an overall low risk of bias, despite a slight penalty in the outcome domain. The study by Thuy et al. (2025) [30] reported a score of 7/9, indicating a moderate risk of bias. In this case, the score was influenced by weaker group comparability. Similarly, the studies by Lim et al. (2017) [20] and Choi et al. (2016) [19], both retrospective cohort studies, achieved a score of 8/9, reflecting a low risk of bias, with minor limitations related to the absence of a control group and restricted comparability.

The risk-of-bias assessment identified some concerns in most randomized trials, particularly regarding selective reporting and, in individual studies, randomization procedures, deviations from the intended interventions, missing outcome data, or outcome measurement. These concerns should be considered when interpreting the findings. The inability to blind participants and clinicians was not considered a source of bias in isolation but was evaluated within the relevant RoB 2 domains.

The certainty of evidence was judged to be low for skeletal transverse changes, dentoalveolar changes, dental tipping, and periodontal and alveolar bone outcomes. The certainty of evidence regarding post-treatment stability and relapse was judged to be very low. Downgrading was mainly related to risk-of-bias concerns, substantial clinical and methodological heterogeneity, indirectness arising from differences in age, skeletal maturity and treatment protocols, imprecision associated with generally small sample sizes, and the limited availability of true long-term post-retention data. The GRADE assessment is summarized in Table 5. The certainty of evidence was low for skeletal transverse, dentoalveolar, and periodontal outcomes and very low for post-treatment stability and relapse.

### 3.1. Skeletal Outcomes

Overall, all expansion protocols produced significant skeletal transverse changes, although the amount and stability of these effects varied according to the type of appliance, patient age, and skeletal maturity.

In pre-pubertal patients, both conventional tooth-borne and skeletal-anchored expanders achieved clinically relevant skeletal expansion. However, skeletal-anchored or tooth–bone-borne appliances generally produced greater initial skeletal effects, particularly in terms of midpalatal suture opening and nasal widening. Bazargani et al. reported greater immediate anterior midpalatal suture opening in the tooth–bone-borne group than in the tooth-borne group (adjusted mean difference: 0.6 mm; 95% CI: 0.2 to 1.1; *p* < 0.01). Nasal width was also greater in the tooth–bone-borne group immediately after expansion (adjusted mean difference: 0.7 mm; 95% CI: 0.1 to 1.4; *p* = 0.03), and the difference increased to 2.1 mm at 5 years (95% CI: 1.4 to 2.8; *p* < 0.01). The difference in anterior sutural opening was no longer present at the 1- and 5-year assessments [27]. Compared with quad-helix treatment, RME produced greater immediate anterior midpalatal suture opening, with adjusted mean differences of 2.9 mm superiorly (95% CI: 2.4 to 3.4) and 3.1 mm inferiorly (95% CI: 2.5 to 3.7; both *p* < 0.001). At the 1-year assessment, the intergroup differences were no longer statistically significant [29]. Paoloni et al. reported greater maxillary skeletal width with RME than with the Leaf expander (Leaf–RME adjusted mean difference: −1.4 mm; 95% CI: −2.4 to −0.3; *p* = 0.013) [25].

Among adolescent patients, skeletal expansion was consistently observed regardless of appliance type. Nevertheless, bone-borne expanders generally produced larger increases in basal maxillary width and zygomatic dimensions than conventional tooth-borne appliances. Thuy et al. reported greater increases in basal maxillary width (+6.79 mm vs. +2.80 mm; *p* = 0.02), zygomatic width (+8.15 mm vs. +4.80 mm; *p* < 0.01), and nasal base width in the bone-borne group [30]. Likewise, both MARPE and conventional RME produced significant increases in skeletal transverse dimensions, including nasal cavity width, anterior nasal cavity width, and posterior nasal cavity width, with most skeletal gains maintained at the end of treatment [24]. Conversely, Kabalan et al. and Kayalar et al. found no significant skeletal differences between bone-borne and tooth-borne approaches in adolescent and surgically assisted expansion protocols, respectively [21,22].

In young adults, where skeletal resistance is expected to be greater, miniscrew-assisted expansion remained effective in producing skeletal widening. Lim et al. reported significant increases in nasal cavity and nasal floor widths, with relapse during follow-up remaining below 1 mm for all skeletal measurements, indicating good long-term stability [20]. Similarly, Choi et al. described a substantial skeletal contribution to total transverse expansion, with most skeletal changes remaining stable during follow-up [19].

### 3.2. Dentoalveolar Outcomes

Significant dentoalveolar changes were consistently reported following maxillary expansion, regardless of patient age or appliance type. In pre-pubertal patients, all appliances produced clinically relevant increases in intermolar and intercanine widths, although the magnitude of dental movement differed according to the anchorage system. Bazargani et al. observed an immediate increase in intermolar width of +5.8 mm in the tooth–bone-borne group and +5.2 mm in the tooth-borne group (*p* = 0.020), with a five-year transverse relapse of −1.1 mm in both groups [27]. Likewise, Paoloni et al. found no significant intergroup difference in intermolar width (Leaf–RME adjusted mean difference: −0.4 mm; 95% CI: −1.2 to 0.5; *p* = 0.365), whereas intercanine width favored RME (Leaf–RME adjusted mean difference: −0.9 mm; 95% CI: −1.5 to −0.3; *p* = 0.005) [25]. Canan et al. also reported significant transverse dental expansion in all groups, with partial relapse during retention but no complete return to baseline values [23].

Among adolescent patients, dentoalveolar expansion remained an important component of treatment despite the greater skeletal contribution observed with bone-borne appliances. Conventional tooth-borne expanders consistently produced greater molar tipping than bone-borne devices. Altieri et al. reported an increase in intermolar angulation of 6.01° in the tooth-borne group compared with only 0.17° in the bone-borne group (*p* = 0.02), together with significantly greater first molar inclination changes [26]. Mehta et al. reported immediate increases in intermolar width of 4.51 mm in the MARPE group (95% CI: 3.46 to 5.55) and 6.07 mm in the RPE group (95% CI: 5.09 to 7.05). Between the post-expansion and final assessments, the corresponding mean changes were +1.10 mm (95% CI: −0.41 to 2.61) and −1.73 mm (95% CI: −3.03 to −0.42), respectively [24]. Periodontal changes also differed between appliances: Altieri et al. demonstrated greater reductions in buccal cortical bone thickness in the tooth-borne group, while Thuy et al. reported only modest reductions in buccal bone thickness after MARPE, with partial recovery during follow-up [26,30].

In young adults, dentoalveolar expansion continued to contribute substantially to total transverse gain. Kayalar et al. found greater anterior dental expansion with tooth-borne appliances than with hybrid expanders (6.13 ± 1.47 mm vs. 4.74 ± 0.79 mm; *p* = 0.019), accompanied by significantly greater molar tipping (9.46° vs. 3.64°; *p* = 0.029) and a greater reduction in buccal alveolar bone thickness [22]. Similarly, Lim et al. reported greater relapse for dentoalveolar than skeletal measurements, with intermolar width decreasing by approximately 2 mm during follow-up, whereas skeletal relapse remained below 1 mm [20].

Overall, dentoalveolar expansion represented an important component of all expansion protocols. Tooth-borne appliances consistently produced greater dental tipping and more pronounced periodontal remodeling, whereas skeletal-anchored devices reduced dentoalveolar compensation while maintaining comparable transverse dental gains. Nevertheless, the magnitude of these differences varied according to patient age, appliance design, retention protocol, and duration of follow-up.

## 4. Discussion

The included studies differed substantially in patient age and skeletal maturity, appliance design, surgical assistance, adjunctive treatment, imaging modality, retention regimen, and follow-up duration. Outcomes recorded during retention were considered maintenance under retention, whereas only post-retention assessments were interpreted as evidence of longer-term stability. To account for this heterogeneity, findings were discussed within four clinically distinct groups: non-surgical expansion in growing or skeletally unstaged patients, non-surgical MARPE or MARME, surgically assisted expansion, and expansion combined with adjunctive orthopedic protocols [19,20,22,25,28,29,30,31].

### 4.1. Non-Surgical Expansion in Growing or Skeletally Unstaged Patients

This group included conventional tooth-borne RPE or RME, non-surgical bone-borne and hybrid expanders, Leaf expanders, and quad-helix appliances in growing or skeletally unstaged patients [21,23,24,25,26,27,29]. Only the RPE arm of Mehta et al. was included in this group [24].

Skeletal outcomes

Tooth-borne, hybrid, and bone-borne protocols produced measurable skeletal widening, although the magnitude and maintenance of these changes differed among appliances and follow-up periods [25,26,27,29].

In growing or skeletally unstaged patients, tooth-borne, hybrid, and bone-borne appliances all produced skeletal widening, although the magnitude and anatomical distribution of expansion varied across studies [25,26,27,29]. Some direct comparisons favored skeletal anchorage at the maxillary base or anterior midpalatal suture, whereas others reported greater widening with conventional RME or no persistent inter-appliance differences. In Bazargani et al., the initial advantage in anterior sutural opening with tooth–bone-borne RME was no longer present at 1 or 5 years, although a difference in nasal width persisted [27]. Therefore, greater initial sutural opening did not necessarily correspond to superior long-term skeletal stability.

Dentoalveolar outcomes

The evaluated protocols increased transverse dental dimensions immediately after active treatment [23,24,25,26,27]. However, the magnitude and distribution of dental expansion differed according to the anchorage system.

In some direct comparisons, tooth-borne appliances produced greater dental displacement or tipping, whereas skeletal anchorage reduced dental effects at selected premolar or molar sites [23,26]. Nevertheless, buccal tipping occurred with different appliance types [23,25,26].

Altieri et al. reported approximately 5 mm of intermolar expansion in both the tooth-borne and bone-borne groups, without significant differences at the coronal or apical levels. However, the intermolar angle increased by 6.01 ± 10.80° with the tooth-borne appliance and remained nearly unchanged with the bone-borne appliance, with a significant intergroup difference (*p* = 0.02) [26]. Tooth-borne anchorage also produced greater molar angulation and greater buccal cortical reduction at the first molars, although significant intergroup differences in cortical thickness were limited to the right buccal side [26]. Thus, the greater skeletal effect of the bone-borne appliance was associated with less inclination but not with greater total dental width.

Canan et al. reported increased transverse dental dimensions and dental tipping in the tooth-borne, bone-borne, and hybrid groups. Partial uprighting occurred during retention, particularly in the hybrid group. Some reduction in dental expansion was observed, although none of the groups returned completely to baseline [23].

Partial posterior uprighting during retention was interpreted as spontaneous realignment rather than complete loss of transverse expansion, particularly after protocols producing greater initial dental inclination [23,27].

Paoloni et al. reported increases in intermolar width of 4.1 ± 1.7 mm with the Leaf expander and 4.5 ± 1.4 mm with RME, without a significant difference. Intercanine width increased by 2.2 ± 1.4 mm and 3.0 ± 1.3 mm, respectively, with a significant difference favoring RME. Changes in molar buccolingual inclination did not differ significantly between treatments [25].

CBCT studies documented reductions in buccal bone thickness and transient periodontal changes after expansion, although the affected appliance and magnitude varied between studies [26,29]. In Hansson et al., post-treatment buccal bone width was lower in the quad-helix group than in the RME group. Buccal fenestrations at the first molars were initially more frequent after quad-helix treatment, but this difference was no longer present at the post-retention follow-up. A marginal bone-level difference persisted only on the right side [29]. Periodontal outcomes therefore did not consistently favor the same appliance across all measurements.

In the RPE arm of Mehta et al., intermolar width increased by 6.07 mm immediately after expansion and decreased by 1.73 mm between the post-expansion and final assessments, while remaining greater than at baseline [24]. Subsequent fixed orthodontic treatment may have influenced the final dental position [24].

Only one study provided prolonged follow-up after appliance removal without additional orthodontic treatment. Bazargani et al. reported identical five-year transverse relapse in the tooth-borne and tooth–bone-borne groups: −1.1 ± 1.2 mm and −1.1 ± 1.1 mm, respectively [27]. Immediate tipping measured 3.4° and 2.5°, whereas five-year angular relapse measured −2.6° and −1.8°. Relapse was mainly attributable to progressive dental uprighting rather than a true loss of transverse dimension [27]. Therefore, although the hybrid appliance produced slightly less initial tipping, it did not demonstrate superior long-term dentoalveolar stability.

Airway findings showed high individual variability and no consistent correlation with skeletal measurements [21]. Kabalan et al. found no significant differences in airway changes among the tooth-borne, bone-borne, and control groups at 6 months and no consistent correlations between airway and skeletal measurements [21]. Respiratory benefits should therefore not be assumed solely on the basis of skeletal widening [21].

### 4.2. Non-Surgical MARPE and MARME Protocols

This group included Choi et al. [19], Lim et al. [20], Thuy et al. [30], and the MARPE arm of Mehta et al. [24]. Choi et al. and Lim et al. included skeletally mature young adults, whereas Thuy et al. included late adolescents and young adults with advanced skeletal maturity. Skeletal maturity was not explicitly staged in Mehta et al. [24], and its results were interpreted with additional caution.

Skeletal outcomes

In skeletally mature or near-mature patients, MARPE and MARME produced measurable skeletal widening, with relatively limited reductions in basal skeletal dimensions during follow-up [19,20,30]. However, the available studies lacked age- and skeletal-maturity-matched tooth-borne control groups, and some final assessments were performed during retention or after subsequent fixed appliance treatment. These findings therefore support the effectiveness and maintenance of miniscrew-assisted expansion but do not establish its superiority over conventional RPE.

Dentoalveolar outcomes

Dentoalveolar changes remained clinically relevant after MARPE or MARME, including molar tipping, reductions in buccal bone thickness, and greater relapse in dental than in basal skeletal dimensions [19,20,30]. Mehta et al. reported different changes in intermolar width after MARPE and RPE; however, all patients subsequently underwent fixed appliance treatment, preventing attribution of the final differences exclusively to expander design [24]. Overall, the available evidence in mature patients mainly reflects short- or mid-term maintenance rather than definitive long-term post-retention stability.

### 4.3. Surgically Assisted Rapid Maxillary Expansion

In surgically assisted expansion, tooth-borne and hybrid appliances produced comparable skeletal widening, whereas the hybrid appliance reduced anterior dental expansion, molar tipping, and selected periodontal effects [22]. However, because the final assessment coincided with appliance removal after 6 months of passive retention, these findings represent maintenance during retention rather than long-term post-retention stability.

### 4.4. Expansion Combined with Adjunctive Orthopedic Protocols

Studies combining expansion with Alt-RAMEC and maxillary protraction were considered separately because their outcomes reflected the combined effects of expansion, constriction, growth, protraction, and skeletal or dental anchorage [28,31]. Both facemask- and mentoplate-based protocols produced sagittal correction, but intergroup differences were generally small or were no longer evident at 5 years. Consequently, these findings cannot be interpreted as isolated evidence of transverse expansion stability.

### 4.5. Overall Interpretation, Clinical Implications, and Limitations

Overall interpretation

Overall, bone-borne and hybrid appliances produced greater initial skeletal changes and fewer dentoalveolar side effects in some direct comparisons, but these advantages were not consistent across measurements, populations, or follow-up periods [22,26,27,30]. A greater initial skeletal contribution did not reliably predict superior long-term stability. Where longer follow-up was available, relapse appeared to involve dental uprighting more frequently than loss of basal skeletal transverse width [20,27].

Clinical implications

Clinically, appliance selection should be individualized according to skeletal maturity, anatomical resistance, periodontal risk, and treatment objectives. Bone-borne or hybrid appliances may be advantageous when reducing dental tipping or buccal bone loss is a priority, whereas tooth-borne expansion remains an appropriate option in growing patients with adequate periodontal support. Current evidence does not support the universal superiority of any expansion appliance.

Limitations

The main limitations of the available evidence were substantial heterogeneity in patient skeletal maturity, appliance design (fixed, removable, and mixed), activation and retention protocols, imaging methods, and follow-up duration; generally small sample sizes; the lack of skeletal-maturity-matched comparisons; the inclusion of adjunctive orthopedic or fixed appliance treatment; and the scarcity of true long-term post-retention observations. The certainty of evidence was low for skeletal, dentoalveolar, and periodontal outcomes and very low for post-treatment stability and relapse. Publication bias could not be formally assessed because no meta-analysis was performed and fewer than 10 studies contributed to each clinically homogeneous comparison. An additional methodological limitation is that PROSPERO registration was completed after the initial database search, which limits the prospective documentation of the review methods.

### 4.6. Comparison with Previous Evidence and Systematic Reviews

Previous systematic reviews have investigated the differences between tooth-borne and bone-borne maxillary expansion approaches, providing findings that are partially consistent with the results of the present review. Krusi et al. compared bone-borne or hybrid tooth–bone-borne rapid maxillary expansion with conventional tooth-borne expansion and reported limited evidence supporting greater skeletal effects with bone-borne appliances, particularly regarding sutural opening; however, no significant differences were observed for dental inclination, nasal cavity width, or root resorption, and the overall quality of evidence remained limited (Krusi et al., 2019) [32]. Similarly, Khosravi et al. evaluated the dentoskeletal effects of tooth-borne versus bone-borne rapid maxillary expansion and concluded that, although bone-anchored devices may provide a more favorable skeletal response, the available evidence was insufficient to demonstrate clear superiority of one expansion modality over another due to methodological heterogeneity and limited study quality (Khosravi et al., 2019) [33]. More recently, a systematic review and meta-analysis focusing on the long-term effects of different expansion appliances reported comparable skeletal and dental expansion outcomes among tooth-borne, hybrid, and bone-borne devices, while suggesting a greater tendency toward molar inclination with tooth-borne appliances (Yu et al., 2026) [34]. Overall, the current evidence suggests that skeletal anchorage may reduce unwanted dento-alveolar effects and enhance orthopedic expansion; however, its superiority in terms of long-term stability remains uncertain. Further well-designed randomized clinical trials with standardized three-dimensional assessment methods and extended follow-up periods are required to determine the most predictable expansion approach.

## 5. Conclusions

Different maxillary expansion protocols can achieve clinically relevant transverse correction. Bone-borne and hybrid appliances may increase the initial skeletal contribution and reduce dentoalveolar side effects, but current evidence does not demonstrate superior long-term stability compared with tooth-borne expansion. Appliance selection, including fixed and mobile devices, should therefore be individualized according to skeletal maturity, anatomical characteristics, and periodontal conditions, considering the limited certainty and scarcity of true long-term post-retention evidence. Currently, there is still no universally accepted and validated orthodontic expansion protocol.

## Figures and Tables

**Table 1:** Search strategy results: databases searched and number of records retrieved.

Date	Database	Query String	Number of Articles
19 October 2025	PubMed	(“Maxillary Expansion”[Mesh] OR “Palatal Expansion Technique”[Mesh] OR “maxillary expansion” OR “palatal expansion” OR “rapid palatal expansion” OR “RPE” OR “MARPE” OR “SARPE” OR “miniscrew-assisted” OR “bone-borne expansion” OR “skeletal expansion” OR “dentoalveolar expansion” OR “tooth-borne expansion”) AND (“stability” OR “relapse” OR “long-term” OR “follow-up” OR “posttreatment” OR “post-treatment” OR “retention” OR “skeletal stability” OR “recurrence”) AND (“suture opening” OR “midpalatal suture” OR “intermolar width” OR “intermolar distance” OR “buccal cortical thickness” OR “bone thickness” OR “alveolar bone” OR “skeletal width”) AND (“humans”[Mesh]) NOT (“case report”[Publication Type] OR “animal study”[tiab] OR “in vitro”[tiab]).	29
		((“maxillary expansion” OR “palatal expansion”) AND (“skeletal” OR “bone-borne” OR “tooth-borne” OR “alveolar”) AND (“stability” OR “relapse” OR “follow-up”))	51
19 October 2025	Scopus	KEY ((“maxillary expansion” AND (“skeletal” OR “alveolar”) AND (“stability” OR “relapse” OR “follow-up”))) AND (LIMIT-TO (SUBJAREA, “DENT”) OR LIMIT-TO (SUBJAREA, “MEDI”)) AND (LIMIT-TO (DOCTYPE, “ar”)) AND (LIMIT-TO (EXACTKEYWORD, “Human”) OR LIMIT-TO (EXACTKEYWORD, “Follow Up”))	14
19 October 2025	Embase	(‘maxillary expansion’/exp OR ‘maxillary expansion’) AND (‘skeletal’ OR ‘alveolar’) AND (‘stability’/exp OR ‘stability’ OR ‘relapse’/exp OR ‘relapse’ OR ‘follow-up’/exp OR ‘follow-up’) AND ([controlled clinical trial]/lim OR [randomized controlled trial]/lim) AND [humans]/lim AND [2015–2025]/py	23

**Table 2:** List of selected studies.

Year	Title	Author	Journal
2015 [21]	Nasal airway changes in bone-borne and tooth-borne rapid maxillary expansion treatments	Ousama Kabalan, Jillian Gordon, Giseon Heo, Manuel O. Lagravere	International Orthodontics
2016 [19]	Nonsurgical miniscrew-assisted rapid maxillary expansion results in acceptable stability in young adults	Sung-Hwan Choi, Kyung-Keun Shi, Jung-Yul Cha, Young-Chel Park, Kee-Joon Lee	Angle Orthodontist
2016 [22]	Comparison of tooth-borne and hybrid devices in surgically assisted rapid maxillary expansion: A randomized clinical cone-beam computed tomography study	Emre Kayalar, Michael Schauseil, Samet Vasfi Kuvat, Ufuk Emekli, Sönmez Fıratlı	Journal of Cranio-Maxillo-Facial Surgery
2017 [23]	Comparison of the treatment effects of different rapid maxillary expansion devices on the maxilla and the mandible. Part 1: Evaluation of dentoalveolar changes	Selin Canan, Neslihan Ebru Senisik	American Journal of Orthodontics and Dentofacial Orthopedics
2017 [20]	Stability of dental, alveolar, and skeletal changes after miniscrew-assisted rapid palatal expansion	Hyun-Mook Lim, Young-Chel Park, Kee-Joon Lee, Kyung-Ho Kim, Yoon Jeong Choi	Korean journal of orthodontics
2022 [24]	Long-term assessment of conventional and mini-screw–assisted rapid palatal expansion on the nasal cavity	Shivam Mehta, Vaibhav Gandhi, Manuel Lagravere Vich, Veerasathpurush Allareddy, Aditya Tadinada, Sumit Yadav	Angle Orthodontist
2022 [25]	Comparison of the dento-skeletal effects produced by Leaf expander versus rapid maxillary expander in prepubertal patients: a two-center randomized controlled trial	Valeria Paoloni, Veronica Giuntini, Roberta Lione, Michele Nieri, Valeria Barone, Matilde Marino Merlo, Francesca Mazza, Stefano Passaleva, Paola Cozza, Lorenzo Franchi	European Journal of Orthodontics
2022 [26]	Comparison of changes in skeletal, dentoalveolar, periodontal, and nasal structures after tooth-borne or bone-borne rapid maxillary expansion: A parallel cohort study	Federica Altieri, Michele Cassetta	American Journal of Orthodontics and Dentofacial Orthopedics
2023 [27]	Three-dimensional comparison of tooth-borne and tooth–bone-borne RME appliances: a randomized controlled trial with 5-year follow-up	Farhan Bazargani, Henrik Lund, Anders Magnuson, Björn Ludwig	European Journal of Orthodontics
2023 [28]	The effect of tooth-borne versus skeletally anchored Alt-RAMEC protocol in early treatment of Class III malocclusion: a single-center randomized clinical trial	Emad Eddin Alzoubi, Simon Camilleri, Mohammed Al Muzian, Nikolai Attard	European Journal of Orthodontics
2024 [29]	Skeletal effects of posterior crossbite treatment with either quad helix or rapid maxillary expansion: a randomized controlled trial with 1-year follow-up	Stina Hansson, Eva Josefsson, Henrik Lund, Silvia Miranda-Bazargani, Anders Magnuson, Rune Lindsten, Farhan Bazargani	Angle Orthodontist
2025 [30]	Clinical and cone-beam computed tomography outcomes of miniscrew-assisted rapid palatal expansion in the treatment of maxillary transverse deficiency A prospective study	Pham Thi Hong Thuy, Pham Thu Trang, Pham Thi Thu Hang, Hoang Viet	Medicine (Baltimore)
2025 [31]	Long-term comparison of maxillary protraction with hybrid Hyrax–facemask vs. hybrid Hyrax–mentoplate protocols using Alt-RAMEC: a 5-year randomized controlled trial	Joeri Meyns, Jeroen Meewis, Flore Dons, Arnoud Schreurs, Johan Aerts, Sohaib Shujaat, Constaninus Politis, Reinhilde Jacobs	European Journal of Orthodontics

**Table 3:** Methodological and clinical characteristics of the included studies.

First Author and Publication Year	Age/Skeletal Maturity	Protocol	Imaging/Measurement Method	Retention Regimen and Follow-Up
Altieri et al. 2022 [26]	Late mixed or permanent dentition; mean age 12.2 years; skeletal maturity not staged	Non-surgical tooth-borne Hyrax vs. miniscrew-supported bone-borne expander	CBCT	Appliances retained for 8 months; assessment at the end of retention; no post-retention follow-up
Alzoubi et al. 2023 [28]	Prepubertal Class III patients, 8–10.99 years; CS1–CS2	Tooth-borne vs. skeletally anchored facemask/Alt-RAMEC	Lateral cephalograms	Assessment at the end of treatment after 9 months; no post-treatment follow-up
Bazargani et al. 2023 [27]	Growing children, 8–13 years; early or late mixed dentition	Non-surgical tooth-borne RME vs. tooth–bone-borne RME	CBCT and dental models	Expander removed after 6 months; follow-up at 1 and 5 years post-expansion
Canan et al. 2017 [23]	Adolescents; mean age approximately 13 years; skeletal maturity not reported	Non-surgical tooth-borne, bone-borne, and hybrid RME	Three-dimensional digital dental models	Six-month retention; assessment at the end of retention only
Choi et al. 2016 [19]	Skeletally mature young adults; mean age 20.9 years; CVMI 6	Non-surgical MARME	Posteroanterior cephalograms and dental casts	Three-month appliance retention followed by fixed treatment; post-treatment follow-up at approximately 30 months
Hansson et al. 2024 [29]	Prepubertal children; mean age 9.5 years; early mixed dentition	Non-surgical RME vs. quad helix	CBCT	Six-month retention; follow-up 1 year after expansion
Kabalan et al. 2015 [21]	Growing patients, 11–17 years; skeletal maturity not reported	Non-surgical tooth-borne Hyrax vs. bone-borne Dresden expander	CBCT + acoustic rhinometry (AR).	Appliances removed and patients reassessed at approximately 6 months; no post-retention follow-up
Kayalar et al. 2016 [22]	Adolescents; skeletal maturity not reported	Surgically assisted tooth-borne Hyrax vs. hybrid Hyrax	CBCT and three-dimensional analysis.	Expander maintained as a passive retainer for 6 months; no orthodontic forces applied during retention.
Lim et al. 2017 [20]	Young adults; mean age 21.6 years; CVMI stage 6, therefore skeletally mature.	Non-surgical MARPE	CBCT	Appliance retained for approximately 4 months; final follow-up approximately 1 year after expansion
Mehta et al. 2022 [24]	Patients aged 11–15 years; mean age 13.69 years in MARPE, 13.9 years in RPE, and 13.3 years in controls; skeletal maturity not explicitly staged.	Non-surgical MARPE vs. tooth-borne RPE vs. untreated controls	CBCT	Retention regimen not clearly reported; final assessment after subsequent fixed orthodontic treatment
Meyns et al. 2025 [31]	Growing skeletal Class III patients; mixed dentition; mean age 9.7 ± 1.3 years.	Hybrid Hyrax–facemask vs. Hybrid Hyrax–mentoplate, both with Alt-RAMEC	Low-dose CT, from which virtual lateral cephalograms were generated.	No separate conventional retention phase is described; protraction therapy continued for about 1 year, and fixed appliances were introduced later in phase 2.
Paoloni et al. 2022 [25]	Prepubertal mixed-dentition patients; CS1–CS2	Non-surgical Leaf expander vs. conventional RME	Digital dental casts, lateral cephalograms, and postero-anterior cephalograms.	In both groups, the expanders were kept as passive retainers for 1 year and removed after one year from application.
Thuy et al. 2025 [30]	Late adolescents and young adults; mean age 20.14 years; CVM stage ≥ 4, indicating advanced skeletal maturity.	Custom-made MARPE	CBCT + lateral cephalometric radiographs.	Appliance kept in place for 6-month retention.

CBCT, cone-beam computed tomography; CVM, cervical vertebral maturation; CVMI, cervical vertebral maturation index; MARME, miniscrew-assisted rapid maxillary expansion; MARPE, miniscrew-assisted rapid palatal expansion; RME, rapid maxillary expansion; Alt-RAMEC, alternate rapid maxillary expansion and constriction.

**Table 4:** Quality of the study detected.

Study	Study Design	Assessment Scale	Score	Quality
Altieri et al. 2022 [26]	Cohort study	Newcastle–Ottawa Scale (NOS)	Selection: 4/4 Comparability: 2/2 Outcome: 2/3	8/9 low risk of bias
Alzoubi et al. 2023 [28]	RCT	RoB 2	Randomization: LR Deviations: LR Missing data: LR Outcome measurement: LR Selective reporting: SC	Some concerns
Bazargani et al. 2023 [27]	RCT	RoB 2	Randomization: LR Deviations: LR Missing data: LR Outcome measurement: LR Selective reporting: SC	Some concerns
Canan et al. 2017 [23]	RCT	RoB 2	Randomization: LR Deviations: LR Missing data: LR Outcome measurement: LR Selective reporting: SC	Some concerns
Choi et al. 2016 [19]	Retrospective cohort study	Newcastle–Ottawa Scale (NOS)	Selection 4/4, Comparability 1/2, Outcome 3/3	8/9 low risk of bias
Hansson et al. 2024 [29]	RCT	RoB 2	Randomization: LR Deviations: LR Missing data: LR Outcome measurement: LR Selective reporting: SC	Some concerns
Kabalan et al. 2015 [21]	RCT	RoB 2	Randomization: SC Deviations: LR Missing data: SC Outcome measurement: LR Selective reporting: SC	Some concerns
Kayalar et al. 2016 [22]	RCT	RoB 2	Randomization: LR Deviations: LR Missing data: LR Outcome measurement: SC Selective reporting: SC	Some concerns
Lim et al. 2017 [20]	Retrospective cohort study	Newcastle–Ottawa Scale (NOS)	Selection 4/4, Comparability 1/2, Outcome 3/3	8/9 low risk of bias
Mehta et al. 2022 [24]	RCT (retrospective analysis)	RoB 2	Randomization: SC Deviations: LR Missing data: LR Outcome measurement: SC Selective reporting: SC	Some concerns
Meyns et al. 2025 [31]	RCT	RoB 2	Randomization: LR Deviations: SC Missing data: SC Outcome measurement: LR Selective reporting: SC	Some concerns
Paoloni et al. 2022 [25]	RCT	RoB 2	Randomization: LR Deviations: LR Missing data: LR Outcome measurement: LR Selective reporting: SC	Some concerns
Thuy et al. 2025 [30]	Prospective study	Newcastle–Ottawa Scale (NOS)	Selection: 4/4 Comparability: 1/2 Outcome: 2/3	7/9 moderate risk of bias

RCT, randomized controlled trial; LR, low risk; SC, some concerns; HR, high risk.

**Table 5:** GRADE.

Outcome Domain	Certainty	Main Reasons for Downgrading
Skeletal transverse changes	Low	Risk-of-bias concerns, heterogeneity, indirectness, and small samples
Dentoalveolar changes and dental tipping	Low	Risk-of-bias concerns, heterogeneous protocols and measurements, and imprecision
Periodontal and alveolar bone changes	Low	Limited studies, heterogeneous measurements, indirectness, and imprecision
Post-treatment stability and relapse	Very low	Risk-of-bias concerns, heterogeneity, imprecision, and scarcity of true long-term post-retention data

## Data Availability

The data presented in this study are available upon reasonable request, after the signature of a formal data-sharing agreement in an anonymous form, from the corresponding author because they are protected by privacy.

## Abbreviations

PRISMA	Preferred Reporting Items for Systematic Reviews and Meta-Analyses
RCT	randomized controlled trial
CBCT	Cone-beam computed tomography
RME	rapid maxillary expansion
MARPE	miniscrew-assisted rapid palatal expansion
TSME	transverse sagittal maxillary expander
PROSPERO	international prospective register of systematic reviews
CCT	controlled clinical trial
RPE	rapid palatal expansion
SARME	surgically assisted rapid maxillary expansion
SARPE	surgically assisted rapid palatal expansion
NOS	Newcastle–Ottawa Scale
FM	facemask
MP	mentoplate
FM/Alt-RAMEC	facemask with alternate rapid maxillary expansion and constriction protocol
TBG	tooth-borne group
BBG	bone-borne group
HG	hybrid expander group
TBB	tooth–bone-borne (hybrid anchorage system)
AMPS	anterior midpalatal suture opening
PMPS	posterior midpalatal suture opening
FMS	frontomaxillary suture
ZMS	zygomaticomaxillary sutures
ANB	angle between point a–nasion–point b (skeletal sagittal relationship)
SNA	sella–nasion–point a angle
SNB	sella–nasion–point b angle
MPA	mandibular plane angle
IMPA	incisor mandibular plane angle
NSDA	nasal septal deviation angle
NH	nasal height
NL	nasal length
NAH	nasal airway height
APL	anterior palatal length
PH	palatal height
U6/L6	upper/lower first molar
U3/L3	upper/lower canine
U1/L1	upper/lower central incisor
U6–U6/L6–L6	intermolar width measurements
U3–U3/L3–L3	interchained width measurements
ICW/ICW4/ICW6	inter-canine width (various tooth levels)
IMW	intermolar width
ABW	alveolar bone width
PNCW/ANCW	posterior/anterior nasal cavity width
Mx–Mx	maxillary width (cephalometric reference points)
PTID	palatal transverse interdental distance
BLI	bucco-lingual inclination
MBR	right mesiobuccal bone plate thickness
DBR	right distobuccal bone plate thickness
MBL	left mesiobuccal bone plate thickness
DBL	left distobuccal bone plate thickness
OL	left oral bone plate thickness
OR	right oral bone plate thickness
BB	bone-borne expander
Alt-RAMEC	alternate rapid maxillary expansion and constriction
